# Metagenome sequencing to analyze the impacts of thiamine supplementation on ruminal fungi in dairy cows fed high-concentrate diets

**DOI:** 10.1186/s13568-018-0680-6

**Published:** 2018-10-03

**Authors:** Fuguang Xue, Xuemei Nan, Fuyu Sun, Xiaohua Pan, Yuming Guo, Linshu Jiang, Benhai Xiong

**Affiliations:** 1 State Key Laboratory of Animal Nutrition, Institute of Animal Science, Chinese Academy of Agricultural Sciences, Beijing, China; 2 State Key Laboratory of Animal Nutrition, College of Animal Science, China Agricultural University, Beijing, China; 3 Beijing Key Laboratory for Dairy Cow Nutrition, Beijing University of Agriculture, Beijing, China

**Keywords:** High-concentrate diet, Metagenome, Ruminal fungi, Thiamine

## Abstract

Ruminal thiamine deficiencies occur when dairy cows are overfed with high-concentrate diet, and thiamine supplementation has been proved to attenuate high-concentrate diet induced SARA. However, there is limited knowledge of the relationship between thiamine supplementation in high-concentrate diets and ruminal fungi. In order to investigate the impacts of thiamine supplementation on ruminal fungi, twelve Chinese Holstein dairy cows were randomly assigned into three treatments: control diet (CON; 20% starch, dry matter basis), high-concentrate diet (HC; 33.2% starch, dry matter basis) and high-concentrate diet supplemented with 180 mg thiamine/kg dry matter intake. Dry matter intake and milk production were recorded during the experimental periods. On day 21, rumen fluid samples were collected at 3 h postfeeding and ruminal pH, thiamine concentration and volatile fatty acids were measured. Metagenome sequencing method was conducted to detect ruminal fungi composition. Feeding HC significantly decreased dry matter intake, milk production, ruminal pH, ruminal acetate and thiamine concentration, however, significantly increased propionate and isovalerate (*P *< 0.05). These changes were inversed by thiamine supplementation (*P *< 0.05). Totally, seven phyla and almost 1050 species of rumen fungi were identified across all samples in which especially, 3 genera and 10 species of strictly anaerobic fungi phylum *Neocallimastigomycota* was found. Principal coordinate analysis indicated that feeding HC and thiamine supplementation caused a significant inverse in ruminal fungi composition. Feeding HC significantly decreased the abundance of fungi compared with CON (P < 0.05) while thiamine supplementation significantly increased the abundance of ruminal fungi (P < 0.05). These results indicated that thiamine supplementation may effectively attenuate rumen metabolic disorder caused by HC diet through buffering the ruminal pH, shifting the rumen fermentation pattern and increasing the abundance of ruminal fungi. The findings in this study could therefore contribute to the further understanding of the mechanism of thiamine’s function in dairy cows.

**Electronic supplementary material:**

The online version of this article (10.1186/s13568-018-0680-6) contains supplementary material, which is available to authorized users.

## Introduction

Ruminal microbiota is a large systematic microbial ecosystem which is composed by an immense variety of bacteria, protozoa, anaerobic fungi and archaea (Hobson and Stewart 1997). Functions of this microbial ecosystem are to digest plant materials and convert them into energy and compounds which are absorbable for ruminants (Fernando et al. 2010; Mao et al. 2016; Pitta et al. 2016). The stability of ruminal microbiota is critical for maintaining the health of ruminants, however, in modern dairy cow production systems, high fermentable carbohydrates are frequently used to obtain higher milk production. The overfeeding of high-concentrate diet decreases ruminal pH, changes ruminal microbiota structure (McCann et al. 2016), influences ruminal fermentation characteristics and consequently leads to subacute ruminal acidosis (SARA) (Enemark 2008). SARA has been reported as one of the most serious nutritional diseases in high yielding dairy cows which costs a considerable economic loss all over the world each year (Valente et al. 2017) and thus many efforts have been paid to prevent its occurrence. Interestingly, our previous study revealed that thiamine supplementation could attenuate high-concentrate diets induced SARA by decreasing ruminal lactate production and increasing ruminal pH value in rumen fluid (Pan et al. 2016). Moreover, ruminal thiamine content was found to be closely related to *Bacteroides, Ruminococcus 1*, *Ruminobacter* etc. Thiamine supplementation to high-concentrate diet attributed to the increasing abundances of these bacteria (Pan et al. 2017). However, these findings mainly focused on ruminal bacterial communities. The information on effect of thiamine supplementation on the composition of other microbial communities including fungi is still limited.

Rumen fungi were first mentioned as a major component of rumen microbiota by a British scientist Colin Orpin (Orpin 1976), which account for about 5% of the total microbial biomass (Rezaeian et al. 2004). In ruminal condition, fungi produced a wide range of enzymes which could digest the major structural carbohydrates of plant cell walls, and ferment diet fiber to product absorbable compounds for the host animal (Fliegerova et al. 2015; Sekhavati et al. 2007). Previous studies about the function of rumen fungi mainly focused on fiber digestion, biogas production (Yıldırım et al. 2017) and carbohydrate degradation (Phillips and Gordon 1995). However, the relationships between thiamine and the rumen fungi are still unclear. Thiamine supplementation shifted the rumen bacterial communities in our previous study (Pan et al. 2017), and thiamine exerts beneficial effects in interactions between fungi and bacteria from lamb rumen (Chaucheyras-Durand and Fonty 2001). Thus, a hypothesis was proposed that thiamine supplementation may affect the abundance and functions of rumen fungi.

In the past years, several molecular techniques including qPCR, Deformation gradient gel electrophoresis (DGGE) and 18S rRNA gene sequences were employed for detecting the predominance and diversity of different genera as well as species of rumen fungi. However, 18S rRNA gene sequencing based analysis of fungi have become obsolete because of the highly conserved nature of the 18S rRNA gene (Fliegerova et al. 2015). Fortunately, based on the development of high through-put sequencing technology, metagenomic sequencing method provided a new way to reveal details about microbial communities (Qin et al. 2013). Metagenomic sequencing mainly uses next-generation sequencing technology to perform shotgun assays on genomic DNA of all microscopic organisms in samples to analyze the genetic diversity and abundance of microbial populations, and to interpret the genetic composition and functional diversity of microbial populations (Kamke et al. 2016; Pitta et al. 2016). Metagenomics sequencing has been widely applied in detecting the diversity and functions of rumen and gut microbiota in recent years and these findings provided us deeper understandings about the micro-ecosystem (Mao et al. 2016; Qin et al. 2014). Therefore, metagenomic sequencing method was used in this study to detect the impacts of thiamine supplementation on ruminal fungi in dairy cows fed high-concentrate diets.

## Materials and methods

### Animals, experimental design and dietary treatments

Animal care and procedures were in accordance with the Chinese guidelines for animal welfare and approved by Animal Care and Use Committee of the Chinese Academy of Agricultural Sciences. Twelve Chinese Holstein dairy cows (627 ± 19.9 kg BW; 180 ± 8 DIM) in second-parity fitted with 10 cm ruminal cannulas (Bar Diamond, Parma, ID) were divided into three treatments. Treatments included a control diet (CON; 20% starch, dry matter basis), a high-concentrate diet (HC; 33.2% starch, dry matter basis) and high-concentrate diet supplemented with 180 mg thiamine/kg dry matter intake (HCT). The diets were formulated according to NRC (2001) to meet or exceed the energy requirements of Holstein dairy cows yielding 20 kg of milk/d with 3.5% milk fat and 3.0% true protein. Details of ingredient analysis and chemical composition of diets were shown in Additional file 1: Table S1. Cows were fed twice equally at 06:00 and 18:00 h each day, and thiamine (thiamine hydrochloride, purity ≥ 99%; Wanrong Science and Technology Development Co., Ltd., Wuhan, China) was add via the rumen cannula twice daily after diets were offered for a 21 day period. Throughout the experimental periods, the cows were housed in individual stalls and had free access to fresh water.

### Rumen fluid sampling and parameters measurement

During the experimental period, automatic feeding equipments (made by Institute of Animal Science Chinese Academy of Agricultural Sciences, Beijing, China and NanShang Husbandry Science and Technology Ltd. Henan, China) were used to record dry matter intake. Milking facilities of Afimilk were applied to record milk production of each cow. On day 21, rumen contents were sampled from cranial, caudal, dorsal, and ventral sites of rumen at 3 h after the morning feeding. Collected samples were strained through four layers of cheesecloth to obtain rumen fluid. Rumen fluid was then divided into two parts. One part was processed to analyze the pH value, thiamine content and rumen volatile fatty acid (VFA) content. The other part was put into the liquid nitrogen immediately after adding stabilizer and then stored at − 80 °C for DNA extraction. Ruminal pH value was measured using a portable type pH meter (Testo 205, Testo AG, Lenzkirch, Germany) immediately after rumen fluid sample collection. Thiamine concentration in rumen fluid was detected by high performance liquid chromatography (HPLC) according to Analytical Methods Committee (2000). Individual and total VFA (TVFA) of ruminal fluid were determined by gas chromatograph (GC-2010, Shimadzu, Kyoto, Japan).

### DNA extraction, library construction, and metagenomics sequencing

DNA for metagenomics was extracted from the rumen fluid samples by using the QIAamp DNA Stool Mini Kit (Qiagen, Hilden, Germany) according to manufacturer’s protocols. The DNA concentration and purity were quantified with TBS-380 and NanoDrop2000, respectively. DNA quality was examined with the 1% agarose gels electrophoresis system.

DNA was fragmented to an average size of 300 bp using Covaris M220 (Gene Company Limited, China) for paired-end library construction. Paired-end library was prepared by using TruSeqTM DNA Sample Prep Kit (Illumina, San Diego, CA, USA). Adapters containing the full complement of sequencing primer hybridization sites were ligated to the Blunt-end fragments. Paired-end sequencing was performed on Illumina HiSeq 4000 platform (Illumina Inc., San Diego, CA, USA) using HiSeq 3000/4000 PE Cluster Kit and HiSeq 3000/4000 SBS Kits according to the manufacturer’s instructions (http://www.illumina.com).

### Sequence quality control and genome assembly

The quality control methods used the following criteria: 3′ and 5′ ends were stripped using SeqPrep (https://github.com/jstjohn/SeqPrep) (Aronesty 2013). Low-quality reads (length < 50 bp or with a quality value < 20 or having N bases) were removed by Sickle (https://github.com/najoshi/sickle). Sequences that lost their mated reads were considered as single reads and were used in the assembly procedure. Reads were aligned to the cow genome by NCBI (https://www.ncbi.nlm.nih.gov) and any hit associated with the reads and their mated reads were removed. Filtered reads were considered for the next step of the analysis.

Considering that K-mers, varying from 1/3 to  2/3 of reads length were used in assembly by SOAP denovo (http://soap.genomics.org.cn, Version 1.06), which is based on De Brujin graph construction (Li et al. 2010). Scaffolds with a length over 500 bp were retained for statistical tests; we evaluated the quality and quantity of scaffolds generated by each assembly and finally chose the best K-mer which yielded the minimum scaffold number and the maximum value of N50 and N90. Then, scaffolds with a length over 500 bp were extracted and broken into contigs without gaps. Contigs were used for further gene prediction and annotation.

### Gene prediction and taxonomy

Open reading frames (ORFs) from each sample were predicted using MetaGene (http://metagene.cb.k.u-tokyo.ac.jp/) (Bao et al. 2014). The predicted ORFs with length being or over 100 bp were retrieved and translated to amino acid sequences using the NCBI translation table (http://www.ncbi.nlm.nih.gov/Taxonomy/taxonomyhome.html/index.cgi?chapter=tgencodes#SG1). All sequences from gene sets with a 95% sequence identity (90% coverage) were clustered as the non-redundant gene catalog by the CD-HIT (http://www.bioinformatics.org/cd-hit/). Reads after quality control were mapped to the representative genes with 95% identity using SOAP aligner (http://soap.genomics.org.cn/) and gene abundance in each sample was evaluated (Qin et al. 2014). DIAMOND (Huson et al. 2016), a new alignment tool for aligning short DNA sequencing reads to a protein reference database was employed for taxonomic annotations by aligning non-redundant gene catalogs against NCBI NR database with e-value cutoff of 1e^−5^ and Score > 60. Based on NCBI Microbial Taxonomy Information Database, species annotation information of genes was obtained and relative abundance of species was calculated at the level of Kingdom, Phylum, Class, Order, Family, Genus and Species.

### Statistical analysis

Ruminal pH and thiamine content were analyzed using one-way ANOVA of SAS 9.2. *P* value < 0.05 was considered to be significance and 0.05 ≤ *P* < 0.10 was considered as a tendency. Spearman correlations between fungi communities and ruminal fermentation variables or thiamine content were assessed using the PROC CORR procedure of SAS 9.2. Relative abundance of all phyla, and the top 30 species of fungi were chosen to conduct the correlation analysis. A correlation matrix was created and visualized in a heatmap format using R package version 3.3.1. The abundance of fungi communities and ruminal variables were considered to be correlated with each other when the absolute value of correlation coefficients (r) were above 0.55 and *P*-value below 0.05. Barplot, principal coordinate analysis (PCoA), hierarchical clustering analysis (HCA) and heat map for different rumen fungi species were conducted using R package version 3.3.1.

### Nucleotide sequence accession number

All the raw sequences were submitted to the NCBI Sequence Read Archive (SRA; http://www.ncbi.nlm.nih.gov/Traces/sra/), under Accession Number SRP144478.

## Results

### Animal performance and rumen fermentation parameters

As shown in Table 1, HC feeding significantly decreased dry matter intake, milk production, ruminal pH value and the concentrations of thiamine and acetate in rumen fluid, while significantly increased the concentration of propionate and isovalerate compared with CON diet (*P* < 0.05). However, these changes caused by HC diet were inversed by thiamine supplementation (*P* < 0.05). Butyrate, valerate, isobutyrate and total VFA concentrations were not affected by dietary treatments (*P* > 0.05).

**Table 1:** Effects of high-concentrate diet feeding and thiamine supplementation on average dry matter intake (DMI), milk production, ruminal pH, ruminal thiamine content and ruminal VFAs content

Item	Experimental treatments			SEM	*P*-value
	CON	HC	HCT		
Average daily DMI (kg/day)	21.68^a^	19.07^c^	20.78^b^	0.278	0.014
Average daily milk production (kg/day)	27.28^a^	22.12^c^	23.28^b^	1.894	0.001
Ruminal pH	6.45^a^	5.58^c^	6.12^b^	0.194	0.016
Ruminal Thiamine (µg/L)	16.16^a^	9.51^c^	13.53^b^	1.933	< 0.001
Acetate (mmol/L)	43.237^a^	42.619^b^	44.076^a^	1.273	0.038
Propionate (mmol/L)	12.899^b^	13.849^a^	11.846^c^	0.632	0.027
Butyrate (mmol/L)	10.770	10.354	10.819	0.137	0.356
Isovalerate (mmol/L)	1.403^b^	1.670^a^	1.366^b^	0.059	0.042
Valerate (mmol/L)	1.212	1.491	1.332	0.059	0.143
Isobutyrate (mmol/L)	0.802	1.001	1.047	0.083	0.504
TVFA (mmol/L)	70.323	70.983	70.487	1.836	0.156

SEM, standard error of the mean; CON, control diet; HC, high-concentrate diet; HCT, high-concentrate diet supplemented with thiamine, TVFA, total volatile fatty acid

^a,b,c^Means within a row with different letters differ significantly (P < 0.05)

### Sequencing information

In the present study, 12 metagenomic libraries were constructed. Based on the quality control methods, all pollution data were removed and approximately 45,000,000 reads of per sample were acquired. The GC content of each sample was between 40–60% except HCT4, which had a lower GC content of 36.36%. The assembly results showed that a minimum of 130,000 contigs per sample were obtained. The N50 value was between 760 and 890 bp for the samples (shown in Table 2). The aligned reads were annotated for gene prediction. The results indicated that numbers of prediction genes were mostly between 200,000 and 400,000. However sample HC3 was detected to contain a significant higher prediction gene numbers while sample HCT4 contained a significant lower prediction gene numbers.

**Table 2:** Quantitative information and quality control of sequencing

Sample	Total reads	Average read length (bp)	Contigs	Prediction genes	N50	N90	GC content (%)
CON1	38,117,710	144.14	136,750	243,399	873	402	49.73
CON2	46,684,704	142.97	150,605	261,343	855	393	49.02
CON3	60,005,524	144.36	205,536	355,099	837	387	48.87
CON4	49,418,758	144.54	185,420	305,814	882	426	51.60
HC1	41,149,472	142.33	130,666	258,388	891	369	50.30
HC2	45,783,576	144.8	164,161	360,929	762	297	46.59
HC3	66,780,112	144.91	266,675	539,128	813	327	47.17
HC4	38,925,364	144.97	140,820	280,129	783	321	47.10
HCT1	51,513,440	144.22	177,350	270,252	897	447	41.29
HCT2	56,614,214	143.39	180,487	283,991	879	423	43.58
HCT3	44,973,212	144.28	161,734	254,637	861	402	41.59
HCT4	38,932,060	143.7	132,493	180,726	867	456	36.36

CON, control diet; HC, high-concentrate diet; HCT, high-concentrate diet supplemented with thiamine

### Effect of HC feeding and thiamine supplementation on relative abundance of fungal communities

**Fig. 1:**
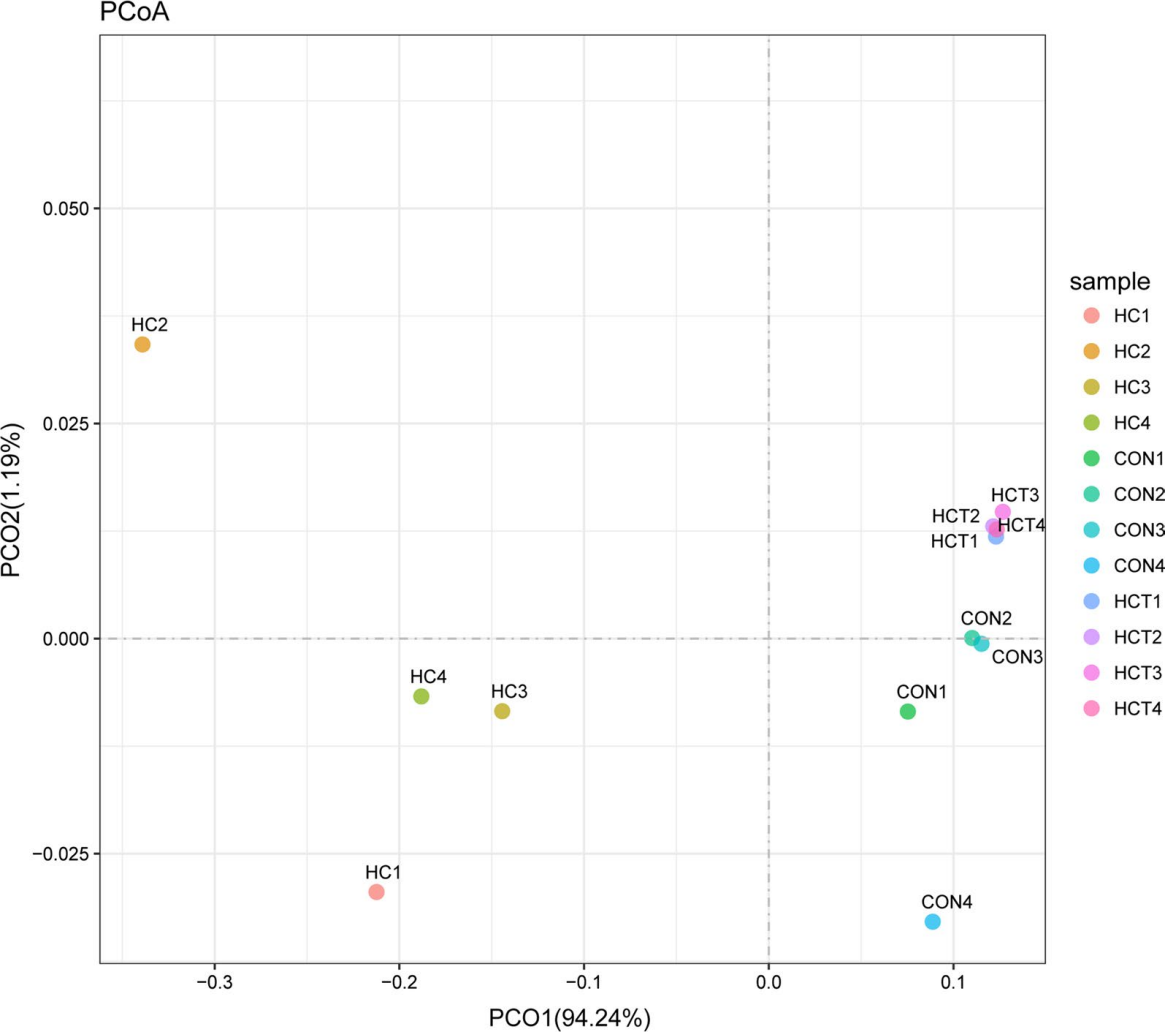
Principal coordinate analysis (PCoA) of fungi community structures of the ruminal fungi in CON, HC and HCT groups. CON, control diet; HC, high-concentrate diet; HCT, high-concentrate diet supplemented with 180 mg thiamine/kg DMI

Principal coordinates analysis (PCoA) based on unweighted UniFrac distance metrics were conducted to compare the three treatments. As shown in Fig. 1, PCoA axes 1 and 2 accounted for 94.24% and 1.19% of the total variation, respectively. The cows offered HC separated from those offered CON and HCT by PCo1 effectively. Similarly, cows fed CON separated from those fed with HCT by PCo2.

**Fig. 2:**
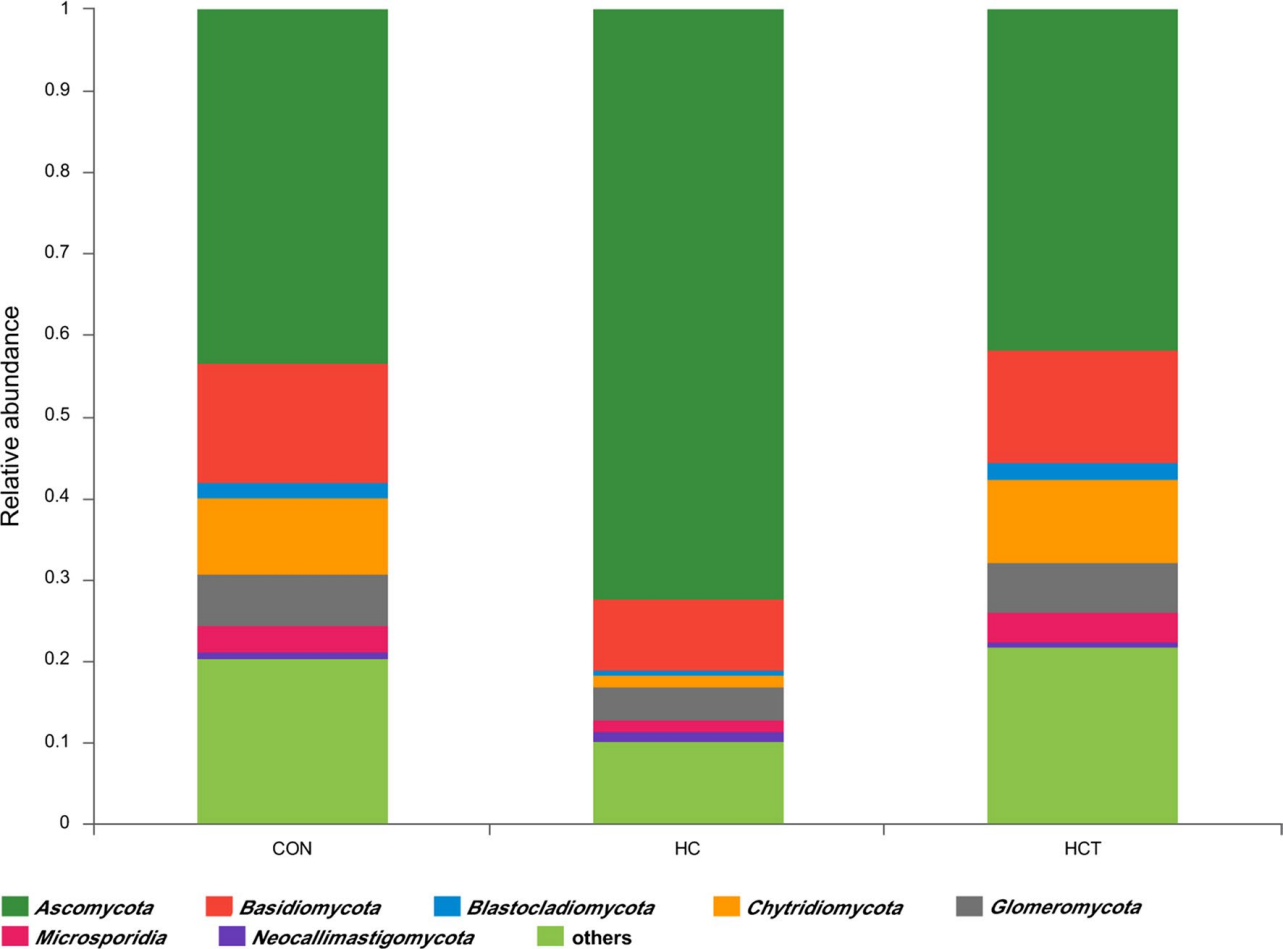
Relative abundance of each ruminal fungi phylum for CON, HC and HCT treatments. CON, control diet; HC, high-concentrate diet; HCT, high-concentrate diet supplemented with 180 mg thiamine/kg DMI

In the present study, approximately 1050 species of rumen fungi were identified across all samples in seven characterized phyla and one uncharacterized phylum. All taxonomy results are shown in Additional file 2: Excel. As shown in Fig. 2, *Ascomycota, Basidiomycota* and *Chytridiomycota* were the three most phyla for all three treatments. Relative abundance of *Ascomycota* was significantly higher in HC treatment than the other two treatments. *Neocallimastigomycota*, characterized as a phylum of strictly anaerobic fungi, was identified including 3 genera (*g__Neocallimastix, g__Orpinomyces,* and *g__Piromyces*) and 11 species (*s__Neocallimastix_frontalis, s__Neocallimastix_patriciarum, s__Orpinomyces_joyonii, s__Orpinomyces_sp._C1A, s__Orpinomyces_sp._OUS1, s__Orpinomyces_sp._PC*-*2, s__Orpinomyces_sp._ukk1, s__Piromyces_equi, s__Piromyces_sp,* and *s__Piromyces_sp._E2*) (shown in Additional file 3). All identified fungi and their relative abundances in the level of phyla are displayed in Table 3. HC feeding significantly decreased the abundance of all phyla of fungi (*P* < 0.05) when compared with CON treatment. In contrast, thiamine supplementation significantly increased the abundance of all phyla of fungi (*P* < 0.05) compared with HC treatment.

**Table 3:** Effect of high-concentrate diet feeding and thiamine supplementation on relative abundances of fungi phylum in rumen fluid (%)

Item	Experimental treatments			SEM	*P*-value
	CON	HC	HCT		
*p__Ascomycota*	0.339^b^	0.107^c^	2.040^a^	0.266	< 0.001
*p__Basidiomycota*	0.116^b^	0.013^c^	0.677^a^	0.090	< 0.001
*p__Blastocladiomycota*	0.014^b^	0.001^c^	0.092^a^	0.012	< 0.001
*p__Chytridiomycota*	0.073^b^	0.002^c^	0.506^a^	0.068	< 0.001
*p__Microsporidia*	0.026^b^	0.002^b^	0.175^a^	0.029	< 0.001
*p__Fungi_noname*	0.130^b^	0.015^c^	0.851^a^	0.114	< 0.001
*p__Glomeromycota*	0.049^b^	0.006^c^	0.294^a^	0.039	< 0.001
*p__Neocallimastigomycota*	0.007^b^	0.002^b^	0.035^a^	0.005	< 0.001

SEM, standard error of the mean; CON, control diet; HC, high-concentrate diet; HCT, high-concentrate diet supplemented with thiamine

^a,b,c^Means within a row with different letters differ significantly (P < 0.05)

**Fig. 3:**
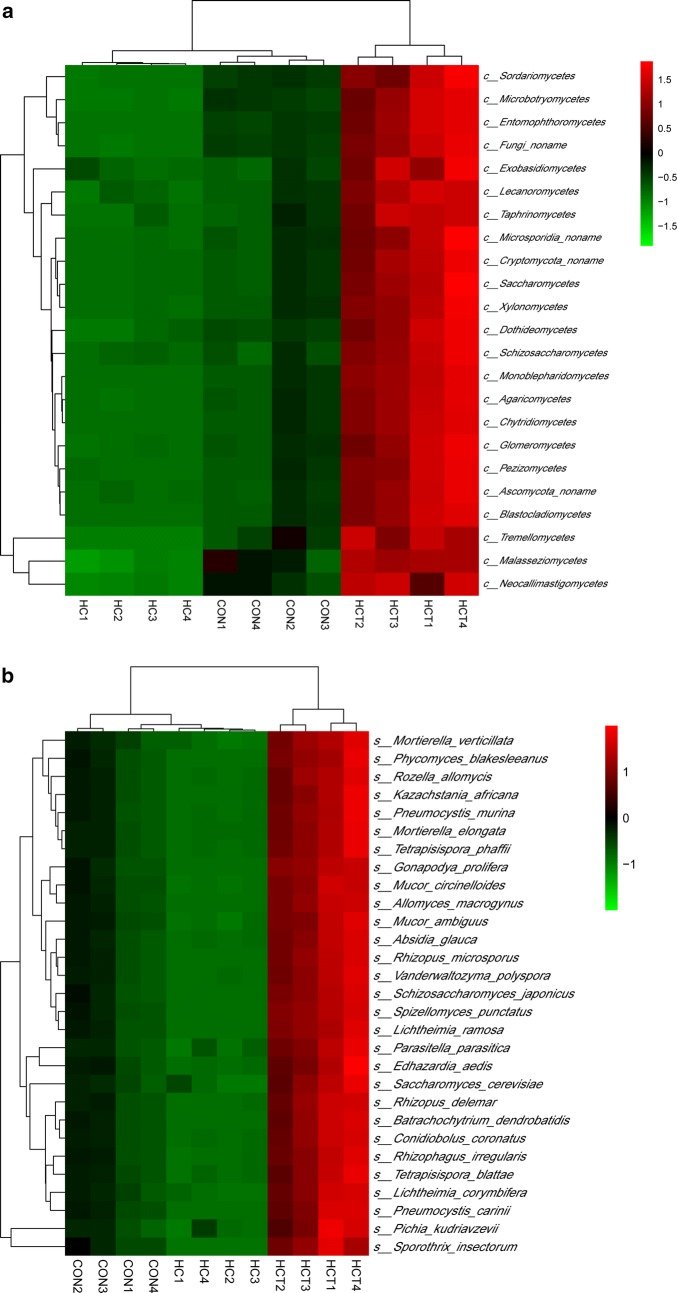
Hierarchical clustering analysis (HCA) and heat map analysis on relative abundances of ruminal fungi content from three treatments: CON, control diet; HC, high-concentrate diet; HCT, high-concentrate diet supplemented with 180 mg thiamine/kg DMI. **a** HCA and heat map analysis on relative abundances of fungi class in rumen fluid; **b** HCA and heat map analysis on top 30 relative abundances of fungi species in rumen fluid. Rows represent fungi and columns represent samples. Cells were colored based on the relative abundance of fungi measured in rumen, red represents high rumen levels while green represents low signal intensity and black cells showing the intermediate level

Hierarchical clustering analysis (HCA) and heat map analysis was conducted to further understand the effects of HC feeding and thiamine supplementation on ruminal fungi profile. Because little information of fungi phyla was detected, taxonomy level of classes and species were used for HCA and heat map analysis. As shown in Fig. 3, samples of HCT treatment gathered into a cluster which was significantly separated from the other two treatments no matter at the level of class or species. Samples of HC treatment were separated from those of CON at the level of class but not at the level of species. Across all three treatments, classes of *Ascomycota, Basidiomycota, Chytridiomycota*, and *Blastocladiomycota* gathered into a big cluster. At the species level, species of *Mucorales* gathered into a big cluster and *Saccharomyces_cerevisiae* which is often used in dairy feed, gathered into a single cluster.

### Correlations between fungi communities and ruminal variables

**Fig. 4:**
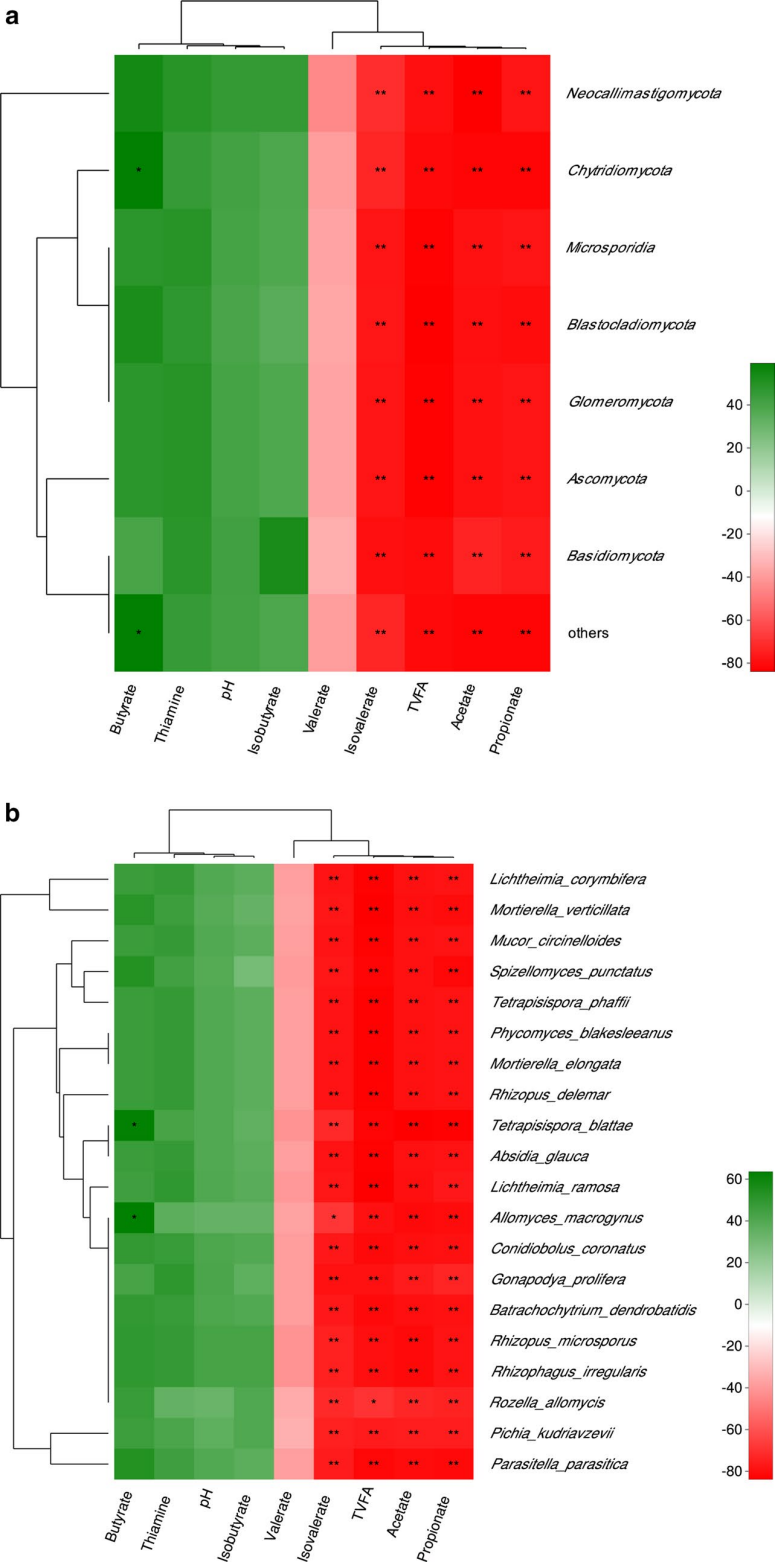
Correlation analyses between relative abundances of fungi and ruminal VFA concentration, thiamine concentrations, and pH. **a** Correlation analyses between relative abundances of fungi phyla and ruminal VFA concentration, thiamine concentrations, and pH. **b** Correlation analyses between relative abundances of fungi species and ruminal VFA concentration, thiamine concentrations, and pH. The red represents a negative correlation, the green color represents a positive correlation. *Represents strong correlation (|r| > 0.55, P < 0.05)

As shown in Fig. 4, the relative abundance of all phyla was negatively correlated with the concentration of TVFA, acetate, propionate and isovalerate (r < − 0.55, *P* < 0.05). In particular, *Chytridiomycota* was positively correlated with butyrate concentration (r > 0.55, *P* < 0.05). There was a trend that the abundances of all phyla of fungi were positively correlated with ruminal pH and thiamine content (r < 0.55, 0.10 > *P* > 0.05). Similarly, all the top 20 species of fungi had significantly negative correlated to TVFA, acetate, propionate and isovalerate (r < − 0.55, *P* < 0.05). Whilst, *Tetrapisispora_blattae* and *Allomyces_macrogynus* both had positively relationships with butyrate content (r > 0.55, *P* < 0.05). In addition, a trend was also found that the abundances of all the top 20 species of fungi were positively correlated with ruminal pH and thiamine content (r < 0.55, 0.10 > *P* > 0.05).

## Discussion

### Composition of ruminal fungi

In the present study 7 phyla and almost 1050 species were identified, the sequencing depth and data size were improved when compared with previous studies (Kumar et al. 2015; Mercado-Garcia 2017; O’Malley et al. 2014), and many species of the rumen fungi were first reported such as *Blastocladiomycota, Chytridiomycota, Microsporidia* and *Glomeromycota* result. The proportion of *Ascomycota* in the whole 7 phyla of fungi in HC treatment significantly increased compared with that in the other two treatments, which indicated that phylum *Ascomycota* had better resistibility to the changed condition caused by HC diet. As the most representative species of *Ascomycota*, *Saccharomyces cerevisiae* was always used as the additive to alleviate subacute ruminal acidosis in lactating dairy cows (Alzahal et al. 2014), and the result further elaborated the function of *Saccharomyces cerevisiae* in alleviating SARA (Alzahal et al. 2014). *Neocallimastigomycota*, although took up a small proportion, attracted more attention in the rumen microbiome (Hibbett et al. 2007) because of its strictly anaerobic characterization and the ability of producing wide array of cell-bound and cell-free cellulolytic, hemicellulolytic, glycolytic, and proteolytic enzymes (Liggenstoffer et al. 2010). Sequencing and functional researches of *Neocallimastigomycota* were proceeded in recent years in order to explore the mechanism of its functions in rumen such as the high-throughput expressed sequence tag (EST) analysis of *Neocallimastix frontalis* (Mi et al. 2009) and the analysis of the *Orpinomyces* genome (Youssef et al. 2013). The results of the present study that the proportion of *Neocallimastigomycota* was increased after thiamine supplementation in the whole rumen micro-biomass may provide useful information in investigating the mechanism of *Neocallimastigomycota.*

### Effect of thiamine supplementation on ruminal fungi composition

In the present study, HC significantly decreased the abundance of fungi (P < 0.05) compared with CON treatment. This finding was in line with several previous studies in which a greater fungal diversity and higher fungal abundance were detected in animals fed high-fiber diets than those offered high-grain diets (Belanche et al. 2012; Kumar et al. 2015). The main function of rumen fungi is to degrade diet fiber and product absorbable compounds for the host animal (Li 2003; Theodorou et al. 1996). Because of the higher gain and lower fiber content in HC, less substrates were provided for fungi growth, therefore leading to the decreased abundance of rumen fungi in HC treatment.

However, the fungal abundance increased significantly after thiamine supplementation in HC treatment which indicated that thiamine supplementation promoted the proliferation and metabolism of rumen fungi. Thiamine is an essential nutrient for dairy cows and plays important roles in carbohydrate metabolism (Kamke et al. 2010; Subramanya et al. 2010). The promotion of carbohydrate metabolism and its interaction with ruminal microbiome might have contribute to the increased fungal abundance.

In ruminal anaerobic environment, ruminal fungi energy generation organelles are represented by hydrogenosomes. These organelles, under anoxic conditions, decarboxylate malate into acetate, CO2 and H2 with concomitant production of energy in the form of ATP (Van 2010; Yarlett and Hackstein 2005). Rumen fungi exhibit tremendous diversity of CAZymes, which work together to degrade carbohydrates and cellulose into monosaccharides and then the monosaccharides are further hydrolyzed into pyruvate (Hall and Mertens 2017). As the cofactor of pyruvate formate-lyase (PFL) and pyruvate:ferredoxin oxidoreductase (PFO), thiamine supplementation promoted the reaction of pyruvate generating into acetyl-CoA (Xue et al. 2018), enhanced the utilization of pyruvate and promoted the degradation efficiency of carbohydrates which lead to more ATP formed to support the metabolism and proliferation of ruminal fungi in the current study. Besides, thiamine supplementation significantly decreased rumen VFAs content and increased ruminal pH (Pan et al. 2016), which promoted the activity of CAZymes (Fliegerova et al. 2015). Therefore, fungal abundance significantly increased in the current study.

Interaction with other microorganisms may also contribute to the change of ruminal fungi. Ruminal microorganism works in harmony to keep the health and productivity of ruminants (Hobson and Stewart 1997; Mickdam et al. 2016). In particular, many positive associations were observed between fungi and bacteria, such as *Caecomyces1*—*Ruminococcus1* and *Piromyces3*—*Succiniclasticum* (Smidt et al. 2010; Tapio et al. 2017). Thiamine was detected to induce a significant increase of fungal growth in fungi and bacteria co-culture system (Shimizu et al. 2016) which indicated that thiamine promoted the mutualism of fungi and bacteria. In addition, previous study of our team indicated that HC significantly decreased the abundance of *Bacteroides* and *Ruminococcus 1* (Pan et al. 2017). As stated above, the decreased *Ruminococcus 1* may partly contribute to the decreased rumen fungi in HC treatment. Thiamine supplementation stimulated the growth of the lactate utilizing bacteria *Ruminococcus 1* (Pan et al. 2017), and thus increased the abundance of rumen fungi indirectly.

In conclusion, HC significantly decreased while thiamine supplementation significantly increased the abundance of rumen fungi. These results indicated that thiamine supplementation may effectively attenuate rumen metabolic disorder caused by HC diet through buffering the ruminal pH, shifting the rumen fermentation pattern and increasing the abundance of ruminal fungi. The findings in this study could therefore contribute to the further understanding of the mechanism of thiamine’s function in dairy cows and provide effective strategies to improve dairy cows’ health under high-concentrate feeding regime.

## Additional files

**Additional file 1: Table S1.** Ingredient and chemical composition of the experimental diets.

**Additional file 2.** Raw data of DMI and milk production.

**Additional file 3.** Taxonomy results of ruminal fungi.
