# Supplementary material for: Metagenome sequencing to analyze the impacts of thiamine supplementation on ruminal fungi in dairy cows fed high-concentrate diets
Source: AMB Express. 2018 Oct 3;8:159. doi: 10.1186/s13568-018-0680-6 (PMC6170517; doi:10.1186/s13568-018-0680-6)
Supplement: Supplementary file 1 — Additional file 1: Table S1. Ingredient and chemical composition of the experimental diets. [file 13568_2018_680_MOESM1_ESM.docx]

AMB Express

Metagenome Sequencing to Analyze the Impacts of Thiamine Supplementation on Ruminal Fungi in Dairy Cows Fed High-Concentrate Diets

***Fuguang Xue ^1,2^******^#^, Xuemei Nan^1#^,Fuyu Sun^1#^ , Xiaohua Pan^1^, Yuming Guo^2^, Linshu Jiang ^3^****^*^* ***and Benhai Xiong^1^****^*^*

^1^ *State Key Laboratory of Animal Nutrition, Institute of Animal Science, Chinese Academy of Agricultural Sciences, Beijing, China, ^2^ State Key Laboratory of Animal Nutrition, Institute of Animal Science, Chinese Agricultural University, Beijing, China, ^3^Beijing Key Laboratory for Dairy Cow Nutrition, Beijing University of Agriculture, Beijing, China*

**^#^** These authors contributed equally to this work.

*^*^***Corresponding author:** Prof. Benhai Xiong, State Key Laboratory of Animal Nutrition, Institute of Animal Science, Chinese Academy of Agricultural Sciences, Beijing 100193, China. Tel: +86-10-62811680; Fax: +86-10-62811680; Email: [xiongbenhai@caas.cn;](%20xiongbenhai@caas.cn;%20)

*^*^***Co-corresponding author:** Prof. Linshu Jiang, Beijing Key Laboratory for Dairy Cow Nutrition, Beijing University of Agriculture, Beijing, China. Tel: +86-10-80798101; Fax:+86-10- 80798101 Email: [jls@bac.edu.cn](%20jls@bac.edu.cn%20)

Table S1. Ingredient and chemical composition of the experimental diets

| Items | CON | SAID |
| --- | --- | --- |
| *Ingredients( % of DM)* |  |  |
| Chinese wildrye | 11.0 | 5.0 |
| Corn silage | 34.0 | 20.0 |
| Alfalfa hay | 15.0 | 15.0 |
| Ground corn | 10.0 | 30.0 |
| Soybean meal, 43% CP | 14.0 | 14.0 |
| Cottonseed meal | 5.0 | 5.0 |
| Distillers dried grains with solubles | 5.0 | 5.0 |
| Whole cottonseed | 3.0 | 3.0 |
| Limestone meal | 1.0 | 1.0 |
| Calcium hydrogen phosphate | 0.7 | 0.7 |
| Sodium chloride | 0.5 | 0.5 |
| Premix^1^ | 0.8 | 0.8 |
| *Nutrient composition (% of DM)* | | |
| NE_L_^2^, Mcal/kg | 1.58 | 1.68 |
| CP | 18.16 | 18.10 |
| Starch | 19.95 | 30.82 |
| NDF | 36.18 | 27.61 |
| ADF | 23.43 | 17.72 |
| NFC^3^ | 32.67 | 45.74 |
| Ether extract | 4.61 | 4.20 |
| Ash | 5.04 | 4.35 |
| Calcium | 0.88 | 0.84 |
| Phosphorus | 0.55 | 0.55 |

Note: 1 Premix contained (per kg): 2142mg of Cu (as sulfate); 15428 mg of Mn (as sulfate); 15428 mg of Zn (as sulfate); 28 mg of Co (as chloride); 231 mg of I (as iodate); 57mg of Se (as selenite); 2285000IU of vitamin A; 457000 IU of vitamin D;11400 mg of vitamin E.

2 NE_L_ was estimated according to NRC (2001).

3 NFC = NFC = 100 – (% NDF + % CP + % ether extract + % ash) (NRC, 2001)
